# Insight from polygenic modifiers in Asian monogenic diabetes

**DOI:** 10.1111/jdi.70289

**Published:** 2026-03-16

**Authors:** Pei‐Ying Kao, Wayne Huey Herng Sheu

**Affiliations:** ^1^ Institute of Molecular and Genomic Medicine, National Health Research Institutes Miaoli Taiwan; ^2^ Division of Endocrinology and Metabolism, Department of Internal Medicine Taichung Veterans General Hospital Taichung Taiwan; ^3^ Division of Endocrinology and Metabolism, Department of Internal Medicine Taipei Veterans General Hospital Taipei Taiwan; ^4^ Department of Internal Medicine Central Clinic and Hospital Taipei Taiwan; ^5^ College of Medicine, National Defense Medical University Taipei Taiwan

## Abstract

A Proposed Translation Roadmap for Asia Diabetes Individuals.

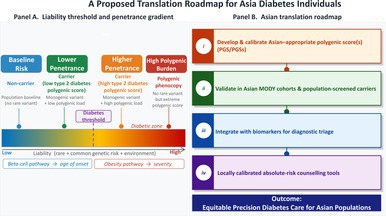

A long‐standing assumption in diabetology is that monogenic diabetes and type 2 diabetes occupy distinct diagnostic boxes, i.e., one driven by rare, high‐impact variants and the other by many common variants of small effect. Yet in clinical practice, especially in Asia, where young‐onset diabetes can present without marked obesity, these boxes frequently overlap. In this timely study, Leech and colleagues^1^ provided unusually quantitative evidence that common genetic variation substantially reshapes disease risk and clinical presentation in hepatocyte nuclear factor (HNF) maturity onset diabetes of the young (MODY), while also highlighting how extreme polygenic burden can mimic MODY‐like phenotypes even when no causal variant was found. Their findings offer a powerful conceptual bridge between “rare” and “common” genetic architectures‐but they also raise an urgent question for Journal of Diabetes Investigation readers: how should such evidence be translated to Asian diabetes individuals?

Leech *et al*.^1^ studied genetically confirmed HNF‐MODY (*HNF1A, HNF4A, HNF1B*) in 1,462 clinically referred cases and compared polygenic score(s) (PGS/PGSs) across several diabetes‐related traits in three groups: HNF‐MODY, individuals with type 2 diabetes, and non‐diabetic controls. Three results stand out. First, HNF‐MODY cases showed strong enrichment of type 2 diabetes polygenic risk (but not type 1 diabetes), supporting a biologically potential model in which polygenic susceptibility to beta‐cell dysfunction modifies the expression of a monogenic beta‐cell disorder. Second, within HNF‐MODY cases, higher PGS of type 2 diabetes was associated with earlier age at diagnosis and greater diabetes severity, consistent with a liability‐threshold framework in which polygenic background influences when and how strongly disease manifests. Third, using a large population cohort, the authors demonstrated a striking penetrance gradient: among carriers of pathogenic variants, diabetes risk ranged from ~11% at the lowest tail of type 2 diabetes PGS to ~81% at the highest tail. In parallel, individuals with MODY‐like clinical phenotypes but no detectable causal variant showed elevated polygenic burden, suggesting “polygenic phenocopy” as a plausible explanation for a subset of MODY‐like presentations.

For Asian diabetes populations, the translational significance is profound but not automatic. A key design feature of the Leech study is that the penetrance gradient in the population cohort was derived from individuals of European ancestry. This is not a limitation of the study's logic, but it is a limitation of direct clinical portability: the distribution of risk alleles, linkage disequilibrium patterns, genetic architecture of type 2 diabetes subtypes, and environmental exposures differ across ancestries. Consequently, PGS thresholds calibrated in Europeans can be systematically mis‐calibrated in East Asians, potentially overestimating risk in some settings and underestimating it in others. If polygenic modifiers are to be used for counseling or diagnostic triage in Asia, they must be reconstructed or recalibrated using ancestry‐appropriate data. In this regard, a large size of population (half millions) derived diseases PGS of Han Chinese Ancestry from Taiwan was recently published.^2^

This also matters because Asia faces a distinct clinical challenge in young‐onset diabetes. Compared with many European‐ancestry settings, East Asian populations often develop type 2 diabetes at lower body mass index (BMI) and with prominent beta‐cell insufficiency.^3^ In daily practice, such presentations can resemble MODY, particularly when autoantibodies are negative and C‐peptide is preserved. The Leech framework suggests two clinically actionable interpretations for this overlap. First, among true MODY carriers, polygenic background may explain why some individuals develop diabetes early and severely while others remain unaffected for decades. Second, among non‐carriers, an extreme polygenic liability for beta‐cell dysfunction could generate a MODY‐like phenotype, creating a “phenocopy” group that meets clinical suspicion criteria but lacks an identifiable monogenic cause. Both scenarios are likely relevant in Asia, where monogenic diabetes remains under‐recognized and access to genetic testing varies widely across countries and health systems.

How should this reshape clinical translation in Asia? We suggest three near‐term priorities. Priority 1: Build and validate ancestry‐appropriate polygenic tools for Asian populations. We need East Asian–calibrated PGSs for type 2 diabetes and relevant traits, evaluated specifically in (i) clinically confirmed MODY cohorts and (ii) population‐screened carriers. This will require large, harmonized Asian datasets and careful calibration to local disease prevalence and ascertainment. Importantly, validation must focus not only on discrimination, but also on calibration: can a given PGS percentile be interpreted as a meaningful shift in absolute risk for an individual patient or family? Priority 2: Use polygenic information to improve diagnostic triage, not to replace monogenic testing. A pragmatic Asian implementation could treat PGS as one additional axis of evidence (alongside age at onset, family history, autoantibodies, C‐peptide, and phenotype) when deciding whom to prioritize for monogenic sequencing and how to interpret ambiguous findings. Priority 3: Redefine penetrance counseling in an Asian context: “variant + background” rather than “variant alone.” In many Asian settings, cascade testing and opportunistic sequencing will increasingly identify pathogenic variants in apparently unaffected individuals. The penetrance gradient shown by Leech *et al*.^1^ illustrates why binary counseling (“you carry it, therefore you will develop diabetes”) is no longer defensible. However, counseling in Asia must be anchored in locally validated risk estimates. Without ancestry‐specific calibration, importing European‐derived penetrance–PGS relationships risks generating false reassurance or undue anxiety. A feasible approach would be to communicate polygenic background as a modifier conceptually, while building the evidence base needed for absolute‐risk counseling in Asian populations.

The Leech^1^ observation that type 1 diabetes polygenic risk is not enriched in HNF‐MODY, whereas type 2 diabetes polygenic risk is enriched, also supports a clinically intuitive use case: aiding differentiation of monogenic diabetes from autoimmune diabetes in young adults, particularly when clinical features are atypical. The goal should be fewer missed MODY diagnoses and fewer unnecessary genetic workups when a polygenic phenocopy is likely. In addition, there might be deeper questions that are particularly relevant in Asia. If polygenic burden modifies MODY expression, then ascertainment bias might become a biological confounder: clinics will preferentially see carriers with both a pathogenic variant and a high polygenic load, while population cohorts will include carriers with lower polygenic liability. This can make penetrance appear “high” in clinical series and “low” in biobanks/populations even when the causal variants are the same. We believe that the impact could be larger in Asia because diagnostic pathways, referral thresholds, and testing availability differ not only by country but also by region and health system.^4^ Bridging these gaps will require prospective studies that combine standardized phenotyping with sequencing and ancestry‐calibrated polygenic modeling. We proposed a translation roadmap for Asian diabetes individuals, shown in Figure 1. Panel A shows a liability‐threshold schematic indicating how a pathogenic monogenic variant shifts baseline liability, while type 2 diabetes polygenic burden modifies penetrance, age of onset, and severity (penetrance gradient). Panel B shows several implementation steps for Asian settings in the hope to reach equitable precision diabetes care for Asian populations.

**Figure 1:**
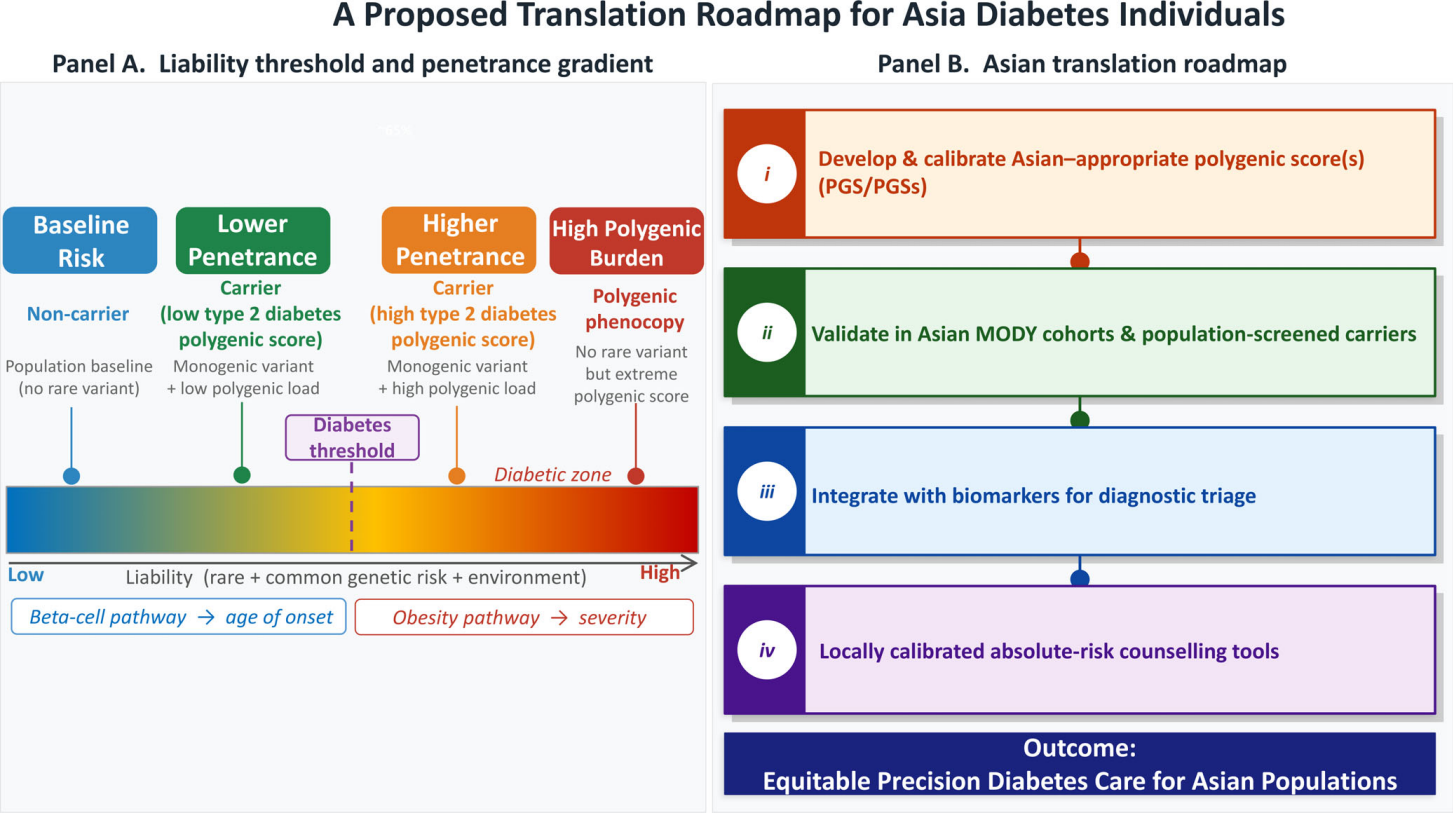
An Asian translation roadmap in young‐onset diabetes. Panel A: Liability‐threshold schematic showing how a pathogenic monogenic variant shifts baseline liability, while type 2 diabetes polygenic burden modifies penetrance, age of onset, and severity (penetrance gradient). Panel B: Implementation steps for Asian settings: (i) develop and calibrate East Asian–appropriate polygenic score; (ii) validate in clinically confirmed maturity onset diabetes of the young cohorts and population‐screened carriers; (iii) integrate with biomarkers (autoantibodies, C‐peptide) and clinical predictors for diagnostic triage; (iv) produce locally calibrated absolute‐risk counseling tools to support cascade testing and follow‐up.

In summary, Leech and colleagues^1^ provide compelling evidence that polygenic background substantially modifies risk and clinical presentation in monogenic diabetes, and that extreme polygenic liability can produce MODY‐like phenotypes without a causal variant. For Asian populations, the key opportunity is not simply to adopt these findings, but to localize them: to build ancestry‐appropriate polygenic tools, to use them as an aid in diagnostic triage and counseling, and to generate Asia‐specific penetrance estimates that reflect local genetics and clinical practice. If achieved, this framework could help the Asian diabetes community reduce missed monogenic diagnoses, avoid overdiagnosis in phenocopies, and move toward truly equitable precision diabetes care.^5^

## Disclosure

The authors declare no conflict of interest.

Approval of the research protocol: N/A.

Informed consent: N/A.

Registry and the registration no. of the study/trial: N/A.

Animal studies: N/A.

## Ethics statement

Not applicable (Commentary; no human/animal subjects).

## Data Availability

Data sharing not applicable to this article as no datasets were generated or analysed during the current study.
